# Energy restriction in obese women suggest linear reduction of hepatic fat content and time-dependent metabolic improvements

**DOI:** 10.1038/s41387-019-0100-2

**Published:** 2019-11-04

**Authors:** Hans-Erik Johansson, David Edholm, Joel Kullberg, Fredrik Rosqvist, Mats Rudling, Sara Straniero, F. Anders Karlsson, Håkan Ahlström, Magnus Sundbom, Ulf Risérus

**Affiliations:** 1 Department of Public Health and Caring Sciences, Clinical Nutrition and Metabolism, Uppsala University, Uppsala, Sweden; 2 Department of Surgical Sciences, Uppsala University, Uppsala, Sweden; 3 Department of Radiology, Oncology and Radiation Science, Uppsala University, Uppsala, Sweden; 4 Department of Medicine, Karolinska Institutet, Stockholm, Sweden; 5 Department of Medical Sciences, Uppsala University, Uppsala, Sweden

**Keywords:** Fat metabolism, Preclinical research

## Abstract

Energy restriction reduces liver fat, improves hepatic insulin resistance and lipid metabolism. However, temporal data in which these metabolic improvements occur and their interplay is incomplete. By performing repeated MRI scans and blood analysis at day 0, 3, 7, 14 and 28 the temporal changes in liver fat and related metabolic factors were assessed at five times during a low-calorie diet (LCD, 800–1100 kcal/day) in ten obese non-diabetic women (BMI 41.7 ± 2.6 kg/m^2^) whereof 6 had NAFLD. Mean weight loss was 7.4 ± 1.2 kg (0.7 kg/day) and liver fat decreased by 51 ± 16%, resulting in only three subjects having NAFLD at day 28. Marked alteration of insulin, NEFA, ALT and 3-hydroxybuturate was evident 3 days after commencing LCD, whereas liver fat showed a moderate but a linear reduction across the 28 days. Other circulating-liver fat markers (e.g. triglycerides, adiponectin, stearoyl-CoA desaturase-1 index, fibroblast growth factor 21) demonstrated modest and variable changes. Marked elevations of NEFA, 3-hydroxybuturate and ALT concentrations occurred until day 14, likely reflecting increased tissue lipolysis, fat oxidation and upregulated hepatic fatty acid oxidation. In summary, these results suggest linear reduction in liver fat, time-specific changes in metabolic markers and insulin resistance in response to energy restriction.

## Introduction

Obesity is closely associated with non-alcoholic fatty liver disease (NAFLD), dyslipidaemia and insulin resistance^1,2^. Among people with NAFLD, ~80% are obese and have type 2 diabetes mellitus (T2DM). Although increased liver fat have been suggested to promote hepatic insulin resistance^3,4^ it is unknown if liver fat reduction precedes improved diet-induced insulin resistance and lipid disorders. Weight loss is the only effective way to reduce liver fat, and also induces multiple metabolic improvements, including enhanced insulin sensitivity. Moderate diet-induced weight loss in obese patients with normal glucose tolerance increase hepatic insulin sensitivity and result in considerable reduction in liver fat by altering multiple mechanisms, e.g. hepatic non-esterified fatty acid (NEFA) uptake and de novo lipogenesis formation with improvements observed within 48 h^5^. Recently, energy restricted diets in the form of low-caloric formula diets (LCD) have received much attention as a potential treatment of T2DM. Lim et al. have reported reversal of T2DM by normalisation of beta cell function and hepatic insulin sensitivity, achieved after only one week of dietary energy restriction. This normalization was associated with decreased liver fat^6^. Little data exist on the temporal course by which these metabolic improvements occur, and how they are inter-related during energy restriction. Hence, the primary aim here was to investigate the temporal changes in liver fat and metabolic markers by magnetic resonance imaging (MRI) scans at day 0, 3, 7, 14 and 28 after commencing a LCD; insulin, alanine aminotransferase (ALT), triglycerides, NEFA, 3-hydroxybutyrate (3-OHB), adiponectin, fibroblast growth factor 21 (FGF-21) and stearoyl-CoA desaturase-1 activity index (SCD), i.e. palmitoleate:palmitate ratio in plasma triglycerides^7–11^.

## Materials and methods

### Study subjects

Patients were recruited from the out-patient clinic for obesity care, University Hospital, Uppsala, Sweden. A total of 10 obese non-diabetic women eligible for bariatric surgery were included in this prospective study. Exclusion criteria were established diabetes, known liver disease, weight above 140 kg or metal implants (the latter two to for MRI compatibility). The study was approved by the regional ethical review board and all patients provided written informed consent.

### Low-calorie diet (LCD)

LCD consisting of Modifast® (Impolin AB, Stockholm, Sweden) was used for 28 days, providing a total energy content of 800–1100 kcal/day (carbohydrates 52%, protein 25% and fat 21%). One meal, one sachet 55 g consists of 214 kcal, fat 4.7 g (0.8 g saturated fat, 1.3 g monounsaturated fat, 2.6 g polyunsaturated fat) carbohydrates 27 g, fibre 4.3 g and salt 0.6 g.

The meal substitute is accordingly European parliament regulation (EU) no. 1169/20121 and directive 96/8/EG.

### Magnetic resonance imaging (MRI)

Liver fat content was assessed by MRI at 1.5 Tesla (Achieva, Philips Healthcare, Best, The Netherlands). Data was analysed by two radiologists and the mean result was used. The MRI methodology used here has been validated against proton magnetic resonance spectroscopy (gold standard)^10^. The clinical procedure and liver fat measurements have been previously described^12^.

### Laboratory analyses

Blood samples were collected, following an overnight fast and fluid consumption was restricted to a total of 500 ml of water during 12 h before examination. Routine blood tests and insulin were analysed at the Department of Clinical Chemistry at University hospital, Uppsala, Sweden. The SCD-index (gas chromatography) was measured at Uppsala University, Sweden. 3-OHB (chromatography-mass spectrometry), adiponectin (sandwich ELISA), NEFA (NEFA-HR (2) photometric assay, Thermo T20xti instrument, Wako Diagnostics, Ca, USA) and FGF-21 (ELISA, human FGF21-kit, Array Reader, BioVendor Research and Diagnostic Products, Brno, Czech Republic) were analysed at Karolinska Institutet, Stockholm, Sweden.

### Clinical measurements

Weight (kg) and height (m) were measured on standardised calibrated scales and BMI (kg/m^2^) was calculated.

### Statistics

All analyses were defined a priori. Results are presented as arithmetic means, with standard deviations. Changes between different time-points were analysed using Student’s tests and Wilcoxson matched pairs. Associations between variables were analysed using Pearson’s product-moment correlation coefficients. Tests were two-tailed and a *p*-value < 0.05 was considered significant. The sample size of ten patients has been shown to have a statistically significant impact of LCD on liver fat and liver volume^12^_._ Statistical software JMP 5.0 for PC (SAS Corporation, Cary, Texas, USA) was used.

## Results

### Baseline data

Patient characteristics, some previously described^12^, at baseline and across the 28-day LCD are shown in Table 1.

**Table 1:** Baseline and follow-up data of 10 morbidly obese women during low-calorie diet for 28 days

Patient Data	Baseline	Day 3	Day 7	Day 14	Day 28	*P* for trend
Age (years)	42.7 (8.9)					
Weight (kg)	114.3 (12.1)	112.4 (11.7)	111.3 (11.9)	110.1 (12.0)	107.0 (11.3)	<0.001
BMI (kg/m^2^)	41.7 (2.6)	41.1 (2.6)	40.7 (2.7)	40.2 (2.8)	39.1(2.6)	<0.001
*MRI data*						
Liver volume (l)	2.1 (0.7)	1.9 (0.7)	1.8 (0.6)	1.7 (0.5)	1.7 (0.5)	<0.001
Liver fat (%)	9.3 (7.1)	8.3 (6.4)	7.3 (5.7)	6.1 (4.5)	4.6 (3.1)	0.006
*Metabolic markers*						
P-glucose (mmol/l)	5.8 (0.6)	5.9 (0.6)	5.9 (0.6)	5.7 (0.6)	5.7 (0.6)	NS
S-insulin (mE/l)	21.4 (9.4)	17.0 (7.2)	16.2 (7.1)	14.8 (4.6)	17.4 (9.3)	0.020
HOMA-IR	5.7 (2.5)	4.5 (2.1)	4.2 (1.9)	3.8 (1.4)	4.6 (3.0)	0.020
P-ALT (μkatal/l)	0.38 (0.10)	0.54 (0.30)	0.67 (0.35)	0.62 (0.21)	0.70 (0.64)	0.013
P-Triglycerides (mmol/l)	2.00 (1.36)	1.85 (1.37)	1.70 (1.03)	1.67 (1.06)	1.45 (0.85)	0.027
P-NEFA (mmol/l)	0.65 (0.15)	0.92 (0.24)	0.88 (0.21)	0.87 (0.21)	0.90 (0.30)	0.005
P-3-OHB (mmol/l)	0.04 (0.05)	0.27 (0.19)	0.25 (0.11)	0.35 (0.25)	0.34 (0.27)	<0.001
P-adiponectin (mg/l)	6.03 (1.95)	6.20 (2.37)	6.28 (2.25)	6.32 (2.11)	6.04 (1.50)	NS
S-FGF-21 (pg/ml)	244 (213)	253 (176)	432 (388)	292 (206)	452 (533)	NS
P-SCD-1 index	3.50 (1.14)	3.34 (1.01)	3.07 (0.94)	3.05 (0.98)	2.97 (0.99)	0.004

Data shown are arithmetic means (±SD)

*BMI* body mass index, *MRI* magnetic resonance imagning, *HOMA-IR* homoeostasis model assessment of insulin resistance, *ALT* alanine aminotransferase, *NEFA* non-esterified free fatty acid, 3-*OHB* 3- hydroxybutyrate, *FGF-21* fibroblast growth factor 21, *SCD-1* stearoyl-CoA desaturase-1 activity index, *P* plasma, *S* serum, *NS* non-significant

### Follow-up data during 28 days of LCD

**Fig. 1:**
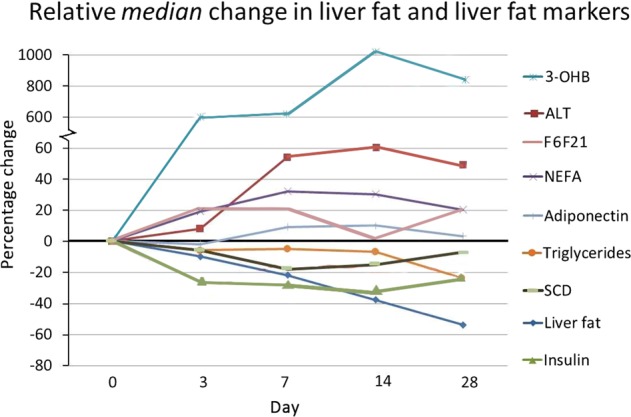
The relative changes during 28 days of low-calorie diet in liver fat content, ALT (alanine transferase), insulin, NEFA, 3-OHB (3-hydroxybutyrate), triglycerides, adiponectin, FGF-21 (fibroblast growth factor 21) and SCD (stearoyl-CoA desaturase-1) activity index in plasma triglycerides. Liver fat decreased linearly with significant change observed from day 3 (11%). Insulin decreased by 23% by day 3 and remained similarly suppressed during the LCD. ALT showed a dramatic increase (53%) by day 7 and remained elevated at day 28. NEFA and 3-OHB showed marked elevations by day 3 and onwards, as expected, whereas triglycerides decreased moderately during the first 14 days followed by further reductions at day 28. FGF-21 showed an initial increase (21% by day 3) with a subsequent plateauing effect

Over the 28-day period there were significant mean changes in weight, BMI, liver fat, liver volume, insulin, HOMA-IR^13^, ALT, triglycerides, NEFA, 3-OHB, SCD as presented in Table 1 and Fig. 1.

### Pearson’s product-moment correlation coefficients

Change in liver fat correlated with change in insulin (*r* = 0.42, *p* = 0.042) and HOMA-IR (*r* = 0.41, *p* = 0.042).

## Discussion

In this study we performed repeated MRI and plasma measurements, to determine time-dependent changes in liver fat and in closely related metabolic markers during energy restriction in a group of obese women. The aim was especially to investigate the early changes in these markers and to follow these over the course of 28 days during energy restriction and diet-induced weight loss. A wide range of biomarkers for liver fat content and NAFLD have been previously measured, mostly in cross-sectional studies but no single or individual marker has been sufficient to evaluate changes in liver fat content^11^. Most likely a cluster of markers are needed for this purpose combined with radiology. Alterations in insulin concentrations have also been studied and they are connected with changes in liver markers and liver fat content^14^. In this study liver fat was the only parameter that showed a linear and stepwise time-dependent reduction during the LCD-diet. NAFLD (liver fat > 5%) was observed in 6 of 10 in the study population at baseline and over the period of 28 days reduced to 3 with the LCD treatment. Thus, more than half of the patients had NAFLD remission. Diet-induced weight loss is an established tool to reduce liver fat and improve glucometabolic status^5^, but little information have been published on how rapid liver fat and liver fat related factors are reduced. The present study is also of current high interest as low-calorie formula diets, as the present one used, are under intense discussion as a promising approach to treat T2DM^6^. In such studies, reduction of liver fat has been proposed as one key improvement that may lead to normalization of hepatic insulin resistance and thereby lowering triglycerides and glucose levels. However, in the present study there were no patients with diabetes, and thus we cannot draw conclusions on the effects on hyperglycaemia as such.

In relative terms, the apparent alterations in insulin and FGF-21 were very rapid and evident already by day 3, whereas the change in liver fat content was slower with only 11% reduction by day 3 (Fig. 1). From these results, one could speculate that liver fat reduction may not necessarily precede the improvements in hepatic insulin resistance, given that fasting insulin, and to some extent also FGF-21, may partly reflect changes in hepatic insulin sensitivity. Such sequence is, however, not supported by experimental data suggesting a causal role of liver fat accumulation on development of hepatic insulin resistance^3^. However, the current reversed scenario with LCD-induced energy deficit and weight loss, not necessarily comparable to the opposite situation with increased liver fat^15^. The increase in ALT concentrations, despite reduced liver fat, may at first glance be surprising, but might reflect high liver exposure to NEFA from lipolysis and upregulated hepatic fatty acid oxidation. An increased tissue lipolysis and fat oxidation occurred until day 14 as indicated by elevated NEFA and 3-OHB concentrations but then levels off. Indeed, these phenomena have been reported in a few previous studies^16–18^. This conclusion was supported by the observation of the parallel dynamic curve of plasma 3-OHB followed that of ALT, which may indicate an association between them. Marked alteration of liver fat, FGF-21 and insulin were observed as early as three days after onset of LCD in these obese women. FGF-21 improves systemic insulin sensitivity and serum FGF-21 levels are increased in obesity, in the starved condition and on exposure to low-protein ketogenic diets^19^ and are independently associated with insulin resistance and metabolic complications^20^. Circulating FGF-21 upregulates adiponectin in subcutaneous adipose tissue and elevated levels of FGF-21 in obesity have been proposed to serve as a defence mechanism to protect against systemic insulin resistance^9^, which may fit with the initial increase in FGF-21 observed at day 3. Interestingly, the response in FGF21 mirrored that of insulin at 3 days, with a subsequent and similar plateauing effect.

The present study has certain limitations. It is a small clinical study involving only 10 obese women, and these metabolic analyses should mainly be regarded as explorative and the findings may need confirmation in studies with larger sample size. However, as far as we know we present novel data regarding short term changes in liver fat and liver fat markers during energy restriction in obese women, evaluated by repeated MRI scans and blood measurements of relevant markers of liver fat as well as glucose and lipid metabolism. The interplay and associations between these and other novel metabolic markers and hepatokines needs to be further studied.

## Conclusion

Energy restriction consistently reduced liver fat content in a linear fashion, but induced pronounced and opposite alterations in fasting insulin and FGF-21 already after 3 days, suggesting rapid improvement of insulin resistance. Markedly elevated NEFA, 3-OHB and ALT concentrations occurred until day 14, possibly reflecting increased tissue lipolysis and fat oxidation. These results suggest linear reduction of liver fat content and time-dependent metabolic improvements in response to short term LCD treatment.
